# Age-related differences in the use of relaxation techniques during intensive professional training

**DOI:** 10.1192/j.eurpsy.2021.1932

**Published:** 2021-08-13

**Authors:** T. Zlokazova, A. Kuznetsova

**Affiliations:** Faculty Of Psychology, Lomonosov Moscow state university, Moscow, Russian Federation

**Keywords:** relaxation techniques, professional training, age differences

## Abstract

**Introduction:**

Intensive professional training is widely used in modern organizations, as it helps employees adapt to dynamic work and technology changes (Noe, 2010; Patrick, 2000). Relaxation techniques may reduce the negative effects of intense learning processes (i.e. fatigue, anxiety and stress). They can also enhance the productivity of the training itself by helping to achieve optimal states for the completion of learning goals.

**Objectives:**

Our study concerns differences in mastering relaxation techniques by employees of younger and middle-age groups during intensive professional training.

**Methods:**

Sample - 62 employees, participants of communication training. The 15-min session of progressive relaxation combined with autogenic formulae was conducted after 5 hours of intensive training. Measures: standard psychological and physiological functional state tests (Leonova & Kapitsa, 2003); an information perception task.

**Results:**

The efficiency of the relaxation techniques varied between different age groups: younger participants (aged 20-30) were more successful in managing both tasks – learning new relaxation skills and achieving deeper rest (including more apparent positive physiological effects). They were also more prepared for completing the information perception task (they made less mistakes). Older participants (aged 30-50) experienced more difficulties with the new relaxation skills and used relaxation primarily to restore their psychophysiological resources, rather than to prepare for the upcoming training task.

**Conclusions:**

The results showed that relaxation techniques provide a system optimization effect on the participants of intensive training programs, though participants’ individual differences (ie age, length of service) should be taken into account when planning the outcomes of such interventions.

**Disclosure:**

No significant relationships.

